# Gambogic acid induces autophagy and combines synergistically with chloroquine to suppress pancreatic cancer by increasing the accumulation of reactive oxygen species

**DOI:** 10.1186/s12935-018-0705-x

**Published:** 2019-01-05

**Authors:** Hongcheng Wang, Zhi Zhao, Shizhou Lei, Shaoli Li, Zhen Xiang, Xiaoyu Wang, Xiuyan Huang, Guanggai Xia, Xinyu Huang

**Affiliations:** 1 Department of General Surgery, Shanghai Jiao Tong University Affiliated Sixth People’s Hospital, 600 Yishan Rd, Shanghai, 200233 China; 2 Department of Gastrointestinal and Hernia Surgery, People’s Hospital of Guilin, Guilin, China; 3 Department of Respiratory Medicine, The First Affiliated Hospital of Sun Yat-sen University, Guangzhou, China; 4 Department of Surgery, Shanghai Key Laboratory for Gastric Neoplasms, Ruijin Hospital, Shanghai Jiao Tong University, Shanghai, China; 5 Department of Endocrinology, Jiangxi Provincial People’s Hospital, Nanchang, China

**Keywords:** Pancreatic cancer, Autophagy, Gambogic acid, Reactive oxygen species, Chloroquine

## Abstract

**Background:**

Gambogic acid is a natural component isolated from gamboge that possesses anticancer properties. Our previous study suggested that gambogic acid might be involved in autophagy; however, its role in pancreatic cancer remained unclear.

**Methods:**

Cell viability and apoptosis of pancreatic cancer cell lines were determined using (4,5-dimethylthiazol-2-yl)-3,5-diphenylformazan and flow cytometry. The effects of gambogic acid on autophagy was assessed by western blot, acridine orange staining, transmission electron microscopy, and measurement of autophagic flux through RFP-GFP-LC3 lentiviral transfection. The mitochondrial membrane potential was assessed by JC-1 staining. The production of reactive oxygen species was measured using CM-H2DCFDA staining. A xenograft tumor model of pancreatic cancer was created to determine the efficacy of gambogic acid and chloroquine.

**Results:**

Gambogic acid induced the expression of LC3-II and Beclin-1 proteins in pancreatic cancer cells, whereas the expression of P62 showed a decline. Gambogic acid also increased the formation of both acidic vesicular organelles and autophagosomes, and increased autophagic flux. These findings indicated that gambogic acid induced the autophagic process. Furthermore, inhibition of autophagy by chloroquine or 3-methyladenine, or knockdown of Atg-7 all enhanced the cytotoxicity of gambogic acid, suggesting that gambogic acid-induced autophagy improves the survival of pancreatic cancer cells. Moreover, gambogic acid reduced the mitochondrial membrane potential and promoted ROS production, which contributed to the activation of autophagy. The inhibition of autophagy by chloroquine further reduced the mitochondrial membrane potential and increased the accumulation of ROS. This indicated that the inhibition of autophagy could mitigate the cellular protective effects induced by gambogic acid. The treatment combination of gambogic acid and chloroquine synergistically inhibited tumor growth in the xenograft tumor model.

**Conclusions:**

These results demonstrate that gambogic acid induces cytoprotective autophagy in pancreatic cancer cells. The inhibition of autophagy promotes the cytotoxicity of gambogic acid by increasing the accumulation of ROS in pancreatic cancer cells. Combining chloroquine and gambogic acid may be a promising treatment for pancreatic cancer.

**Electronic supplementary material:**

The online version of this article (10.1186/s12935-018-0705-x) contains supplementary material, which is available to authorized users.

## Background

Pancreatic cancer is one of the most malignant tumors, and the fourth leading cause of cancer-related deaths worldwide. Although early diagnosis and treatment with surgery and chemotherapy improve the prognosis of pancreatic cancer, the 5-year survival rate is less than 5% [1]. Moreover, Pancreatic cancer is often resistant to chemotherapy and radiotherapy [2, 3]. Therefore, exploration of a novel systemic treatment is urgently needed.

Macroautophagy (hereafter referred to as autophagy) is a highly conserved cellular process, which entails the capture and digestion of proteins and organelles to provide energy and eliminate damaged organelles. Autophagy assists in overcoming metabolic stress and maintaining cellular homeostasis [4, 5]. It has been observed in a variety of cancers, and its functions differ depending on the type of cancer [6–8]. Previous studies have reported that autophagy is not only protumorigenic, but also tumor suppressive during the progression of pancreatic cancer [9–11]. However, an increasing number of studies show that autophagy plays a cytoprotective role in pancreatic cancer. Recent studies have demonstrated that the inhibition of autophagy can promote the sensitivity of pancreatic cancer cells to chemotherapy [12–15]. Thus, the combination of an autophagy inhibitor with chemotherapy may be a promising strategy to improve survival in patients with pancreatic cancer.

Gambogic acid (GA) is one of the main components isolated from gamboge that reportedly possesses proapoptotic activity in various types of cancer [16]. It induces apoptosis both directly by activating the caspase pathway, and indirectly by inducing stress in pancreatic cancer cells [17, 18]. It also induces autophagy in various cancers. However, whether GA induces autophagy in pancreatic cancer is unknown [17, 19]. In this study, our results demonstrated that GA induces the autophagic process in pancreatic cancer, and this confers cytoprotection and promotes the survival of pancreatic cancer cells. Furthermore, inhibition of autophagy with chloroquine (CQ) effectively promotes the cytotoxicity of GA against pancreatic cancer cells in vitro and in vivo.

## Materials and methods

### Reagents and cell lines

Gambogic acid (98% purity), CQ, acridine orange, and (4,5-dimethylthiazol-2-yl)-3,5-diphenylformazan (MTT), were purchased from Yuanye biotech (China). Both 3-methyladenine (3-MA) and bafilomycin A1 (Baf-A1) were purchased from Selleck Chemicals (USA). An Annexin V/PI apoptosis kit was purchased from Vazyme (China). Primary antibodies against cleaved-PARP, cleaved caspase-3, cleaved caspase-9, bcl-2, mTOR, and phospho-mTOR were purchased from CST (Cell Signaling Technology, USA). Beclin-1, P62, and LC-3 were purchased form ProteinTech (USA). An IHC (immunohistochemical) detection kit was purchased from CWBio (China). Human pancreatic cancer cell lines PANC-1 and BxPC-3 were purchased from the Type Culture Collection of the Chinese Academy of Sciences (Shanghai, China). The BxPC-3 cells were cultured in Roswell Park Memorial Institute medium (RMPI 1640) supplemented with 10% FBS (fetal bovine serum), and PANC-1 cell lines were cultured in Dulbecco’s modified Eagle’s medium (DMEM) supplemented with 10% FBS. All cell lines were maintained in an incubator at 37 °C with 5% CO_2_.

### Western blotting

Cells (1 × 106) were seeded in 60 mm^3^ dishes and cultured overnight. After being treated different reagents according to this study, total protein was extracted. Total protein was then extracted with radioimmunoprecipitation assay (RIPA) buffer (Beyotime Biotechnology, China) containing 1% phenylmethylsulfonyl fluoride (PMSF) (Beyotime Biotechnology China). Protein concentration was detected, and similar quantities of protein were used for western blot analysis. Total protein was separated by sodium dodecyl sulfate-polyacrylamide gel electrophoresis (SDS-PAGE) (10–15%), and electrically transferred onto polyvinylidene difluoride (PVDF) membranes. The membranes were blocked with 5% defatted milk for 1 h at room temperature, incubated with the relevant primary antibody overnight at 4 °C, and then washed three times with 0.1% TBST [Tris-buffered saline (TBS) and Tween 20], for 7 min each time. Membranes were incubated with second antibody for another 1 h at room temperature. The washing process was repeated and the immunoreactive bands were detected using an enhanced chemiluminescence (ECL) reagent (Thermo Fisher Scientific, USA).

### Acridine orange staining

The formation of acidic vesicular organelles (AVOs) within cells provided basic evidence of autophagy. In addition, AVOs were detected by acridine orange staining. Following treatment, PANC-1 and BxPC-3 cells were stained with 1 µg/mL of acridine orange for 20 min at 37 °C, and cells were then washed with phosphate-buffered saline (PBS) twice. The AVOs were detected using a fluorescence microscope (Olympus IX70), and the cells with red fluorescence were counted in six random fields.

### Measurement of autophagic flux

The PANC-1 cells (2 × 10^4^) were seeded into 12-well plates and incubated overnight. The cells were then transfected with RFP-GFP-LC3 lentivirus (Gene Pharma, China) using polybrene. After incubation for 2 days, cells were observed under a fluorescence microscope. Cells were then augmented and cryopreserved for further study. The PANC-1 cells transfected with RFP-GFP-LC3 were seeded on glass coverslips. The fluorescence of cells was subsequently detected using an Olympus IX70 microscope, and the average numbers of autophagosomes (yellow dots) and autolysosomes (red dots) within cells were counted.

### Transmission electron microscopy

After being treated with GA, PANC-1 cells were fixed with 2.5% glutaraldehyde containing 0.1 M sodium cacodylate, and subsequently fixed in 1% phosphate-buffered osmium tetroxide, and stained with 3% aqueous uranyl acetate. Samples were then dehydrated through a graded series of ethanol and subsequently embedded. After being sectioned, samples were stained with lead citrate, and examined under a Philips EM420 transmission electron microscope.

### MTT assay and combination index

Cell viability was detected using the MTT assay. Cells (8 × 103) were seeded into 96-well culture plates for incubation overnight. The culture medium was then discarded, and 100 µL of the culture medium containing 20% MTT solution was added into the wells for incubation over 4 h at 37 °C. The MTT solution was then aspirated, and 150 μL dimethyl sulfoxide (DMSO) solution was added into the wells, which were then incubated at 37 °C for 15 min. The sample was then shaken, after which the absorbance was measured at 490 nm using a microplate reader. For combined treatment with two drugs, cells were first exposed to CQ for 24 h, then treated with GA for another 24 h after washout of CQ. The combination index (CI) was calculated according to the method of Chou and Talalay, using the Calcusyn software (Biosoft, UK). If the CI < 0.90, this indicated synergism; a CI between 0.90 and 1.10 indicated an additive effect; and a CI > 1.10 indicated antagonism.

### Apoptosis assay

The PANC-1 and BxPC-3 cells were seeded into 60 cm^2^ dishes. After the cells reached 70–80% confluence, they were treated with either GA or CQ for 24 h. For the combined treatment, cells were cultured with CQ for 24 h, and subsequently incubated with GA for another 24 h. The cells were then digested with trypsin and collected. Annexin V and PI solutions were used to dye the cells for 10 min in the dark, and a flow cytometer (Beckman, Navios 2L 8C, USA) was used to detect the apoptotic cells. Data were analyzed by the FlowJo V10 software.

### Xenograft tumor model

All animal experiments were approved by the Ethical Review Committee of The Six Affiliated Hospital of Shanghai Jiaotong University. The 6–8 weeks old, BALB/c female nude mice were purchased from Shanghai Si Lai Ke Laboratory Animal Co. Ltd, China. Xenograft tumor models were created by subcutaneously injecting 5 × 10^6^ BxPC-3 cells into the right flank of the mice. After tumors grew to 40 mm^3^, mice were randomly divided into four groups (n = 5) as follows: the control group (treated with saline); GA group (8 mg/kg, once every 3 days); CQ group (100 mg/kg, once every 3 days); and the combination group (first day treatment with 100 mg/kg CQ, second day treatment with 8 mg/kg GA, with an interval of 3 days between each treatment). Tumor size was measured and tumor volume was calculated using the formula: volume = 0.5 × (length × width^2^). After 27 days, mice were euthanized and xenograft tumors were collected and weighed.

### Immunohistochemical (IHC) analysis

Paraffin-embedded xenograft tissues were cut into 4 µm-thick slices. Tumor sections were deparaffinized, rehydrated, and antigen-retrieved with citric acid. The sections were then blocked with goat serum for 1 h at room temperature, and incubated with primary antibodies overnight at 4 °C. Tissue sections were then further incubated with horseradish peroxidase (HRP)-conjugated second antibody for 30 min at 37 °C. After routine washing, slices were counterstained with hematoxylin. The antigen was detected using diaminobenzidine (DAB) solution.

The IHC results were observed under a microscope, and IHC evaluation of Ki-67 and terminal deoxynucleotidyl transferase dUTP nick end labeling (TUNEL) was based on the percentage of positive cells. The score of staining intensity ranged from 0 to 3 points (0, absent; 1, weak; 2, moderate; and 3, intense). The score of staining proportion ranged from 1 to 3 points (1, < 10%; 2, 10–49%; 3, > 50% of positive cells), and the IHC score was calculated by multiplying the two scores. Cells in five random fields under 400× magnification were counted. Quantification of IHC was performed according to the methods of previous studies [20, 21].

### Measurement of ROS

After designated treatment, the PANC-1 and BxPC-3 cells were collected, washed with PBS three times, and counted on a hemocytometer. Cells were resuspended in 1 mL serum-free medium, and stained with 0.1 mM dichlorodihydrofluorescein diacetate (DCFH-DA) (Beyotime Biotechnology, China) for 30 min at 37 °C. The cells were then washed three times with serum-free medium, and fluorescence was examined using a fluorescence microplate (excitation wavelength: 488 nm; emission wavelength: 525 nm). The level of fluorescence was used to indicate ROS levels, and data were displayed using the fold change in fluorescence.

The PANC-1 and BxPC-3 cells were then seeded into 6-well plates. Cells were washed with PBS, and incubated with serum-free medium containing 0.1 mM DCFH-DA for 30 min at 37 °C. They were then washed with serum-free medium three times, and fluorescence was detected and captured using a fluorescence microscope.

### Measurement of MMP (mitochondrial membrane potential)

The mitochondrial membrane potential of pancreatic cancer cells was detected by the JC-1 assay. The PANC-1 and BxPC-3 cells were seeded into 6-well plates. After being treated, cells were collected and resuspended in the culture medium. Cells were then stained with the JC-1 working solution (Beyotime Biotechnology, China) for 20 min at 37 °C, and reverse-blended twice during staining. After staining, cells were washed three times with JC-1 buffer solution, and centrifuged at 600 rcf (relative centrifugal force) for 5 min. The mitochondrial membrane potential was then examined using flow cytometry (excitation wavelength: 490 nm; emission wavelength: 530 nm), and the data were analyzed by the Kaluza for Gallios software (USA).

### Statistical analysis

All statistical analyses were performed using the SPSS 18.0 software. Data were presented as mean ± SD. The Student’s *t*-test was used to calculate the *P*-value. Two-sided *P*-values < 0.05 were considered statistically significant.

## Results

### GA induces the autophagic process in pancreatic cancer cells

**Fig. 1:**
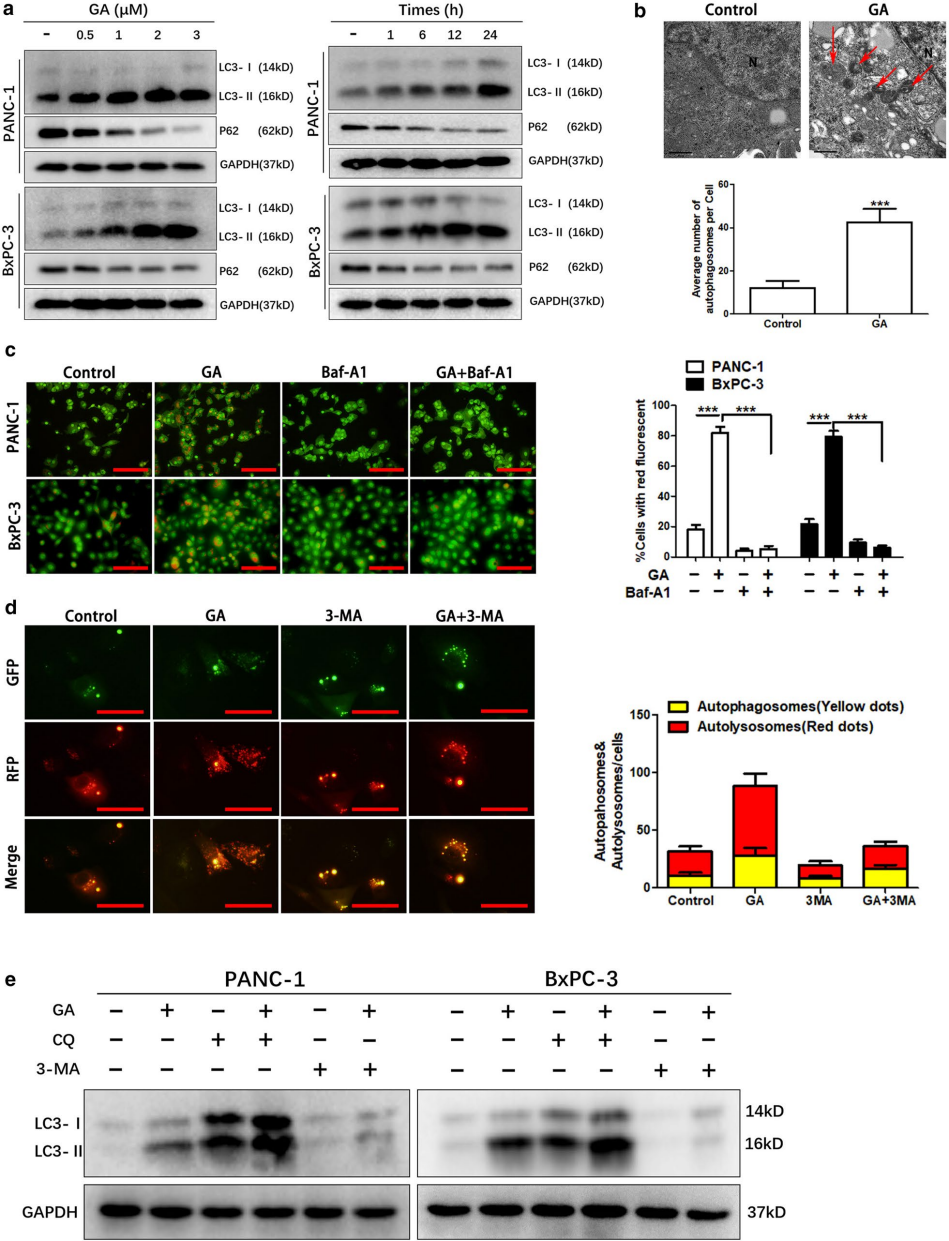
Gambogic acid (GA) induces autophagy in pancreatic cancer cells. **a** PANC-1 and BxPC-3 cells were treated with the indicated concentrations of GA for 24 h, or with 1 µM GA for the indicated times. The expression of LC3 and P62 proteins was analyzed by western blot. **b** PANC-1 cells were treated with 1 µM GA for 24 h, and transmission electron microscopy was used to visualize autophagic vacuoles. **c** PANC-1 and BxPC-3 cells were treated with 1 µM GA in either the absence or presence of bafilomycin A1 (Baf-A1) (100 nM) for 24 h. Acidic vesicular organelles (AVOs) were detected with acridine staining, and the number of cells with AVOs was quantified. Scale bars indicate 200 µm. **d** Stable GFP-mRFP-LC3 transfected PANC-1 cells were treated with 1 µM GA in the absence or presence of 3-methyladenine (3-MA) (10 mM) for 24 h, and cells were observed under a fluorescence microscope. The number of autophagosomes (yellow dots) and autolysosomes (red dots) within each cell were counted; n = 50 cells/sample. **e** PANC-1 and BxPC-3 cells were treated with 1 µM GA in the absence or presence of 3-MA (10 mM), or chloroquine (CQ) (40 µM) for 24 h. The LC3 protein was analyzed with western blot. Data are presented as mean ± SD (n = 3); *** indicates *P *< 0.001

Autophagy reportedly plays a divergent role in pancreatic cancer, and is associated with apoptosis [10]. Our previous study reported that GA induces the apoptosis of pancreatic cancer cells [18]. To investigate whether GA induces autophagy, we analyzed the conversion of LC3-I to LC3-II by immunoblot, which confirmed the occurrence of autophagy. The results revealed that GA increased the ratios of LC3-II to LC3-I in a dose- and time-dependent manner. Our findings also showed that GA increases the clearance of P62, which is another feature of autophagy (Fig. 1a). Transmission electron microscopy revealed that GA increased the number of autophagic vacuoles in PANC-1 cells (Fig. 1b). As the formation of AVOs is an established feature of autophagic cells, we performed acridine staining to detect this phenomenon in PANC-1 and BxPC-3 cells treated with GA (Fig. 1c). The results showed that GA evidently increased the formation of AVOs, and this action was inhibited when the cells were co-treated with the autophagy inhibitor Baf-A1 (bafilomycin-A1), indicating that GA promoted the accumulation of autophagosomes.

Fusion with lysosomes contributes to the maturation of autophagosomes, and the inhibition of this process impairs autophagic degradation [4]. To investigate whether GA promotes autophagy by impairment of autophagosome maturation, we detected autophagic flux in PANC-1 cells with tandem-labeled GFP-mRFP-LC3. The RFP fluorescence was detected in autophagosomes and autolysosomes, whereas GFP fluorescence was quenched in autolysosomes owing to their acidic environment. Therefore, when autophagic flux exists within cells, the fusion of lysosome and autophagosome would cause a reduction in yellow fluorescence and increase in red fluorescence. As shown in Fig. 1d, GA significantly increased RFP fluorescence and reduced the yellow fluorescence, and RFP fluorescence was reduced when PANC-1 cells were co-treated with both the autophagy inhibitor 3-MA and GA. We also found that the lysosomal inhibitor and autophagy inhibitor, CQ, could increase the accumulation of LC3-II in PANC-1 and BxPC-3 cells when used as a co-treatment with GA. In addition, GA-induced LC3-II accumulation could be impaired by 3-MA (Fig. 1e). These results demonstrate that GA induces autophagy in pancreatic cancer cells.

### GA-induced autophagy is cytoprotective in pancreatic cancer cells

**Fig. 2:**
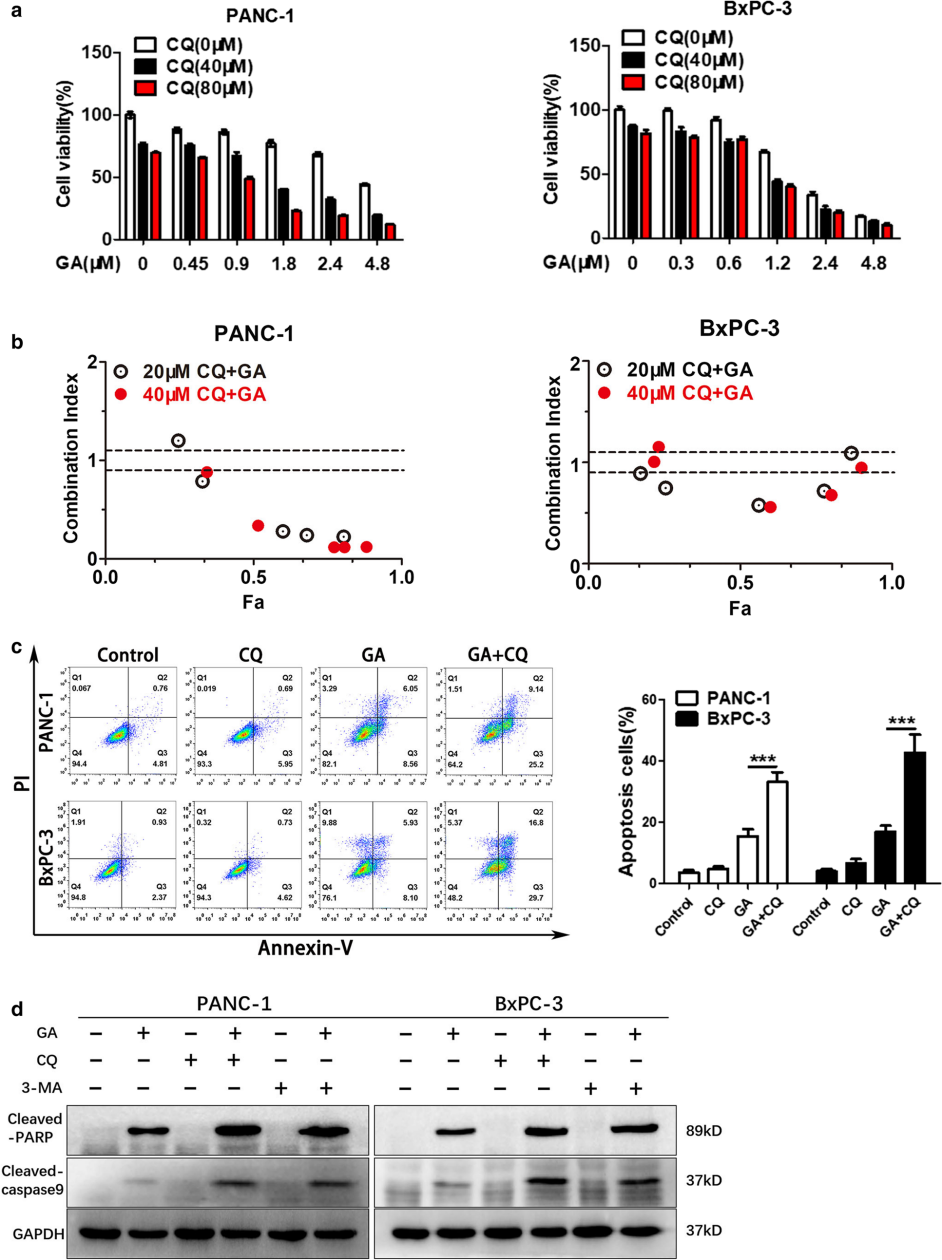
Inhibition of autophagy sensitizes pancreatic cancer cells to gambogic acid (GA). **a** PANC-1 and BxPC-3 cells were pretreated with the indicated concentrations of chloroquine (CQ) for 24 h, and then treated with the indicated concentrations of GA for another 24 h. Cell viability was detected with the MTT assay. **b** The combination index (CI) for PANC-1 and BxPC-3 cells was calculated using the Chou–Talalay method and CalcuSyn software. ‘‘Fa’’ refers to the inhibitory rate. CI < 0.90 indicates synergism; a CI between 0.90 and 1.10 indicates an additive effect; CI > 1.10 indicates antagonism. **c** PANC-1 and BxPC-3 cells were pretreated with CQ (40 µM) for 24 h, and then treated with GA (1 µM) for another 24 h. Apoptosis was detected using the Annexin V/PI double stain, and flow cytometry was performed. **d** PANC-1 and BxPC-3 cells were treated with 2 µM GA in the absence or presence of 3-methyladenine (3-MA) (10 mM), or CQ (40 µM) for 24 h. The expression of cleaved caspase-9 and cleaved-PARP protein was analyzed with western blot. Data are presented as mean ± SD (n = 3); *** indicates *P *< 0.001

**Fig. 3:**
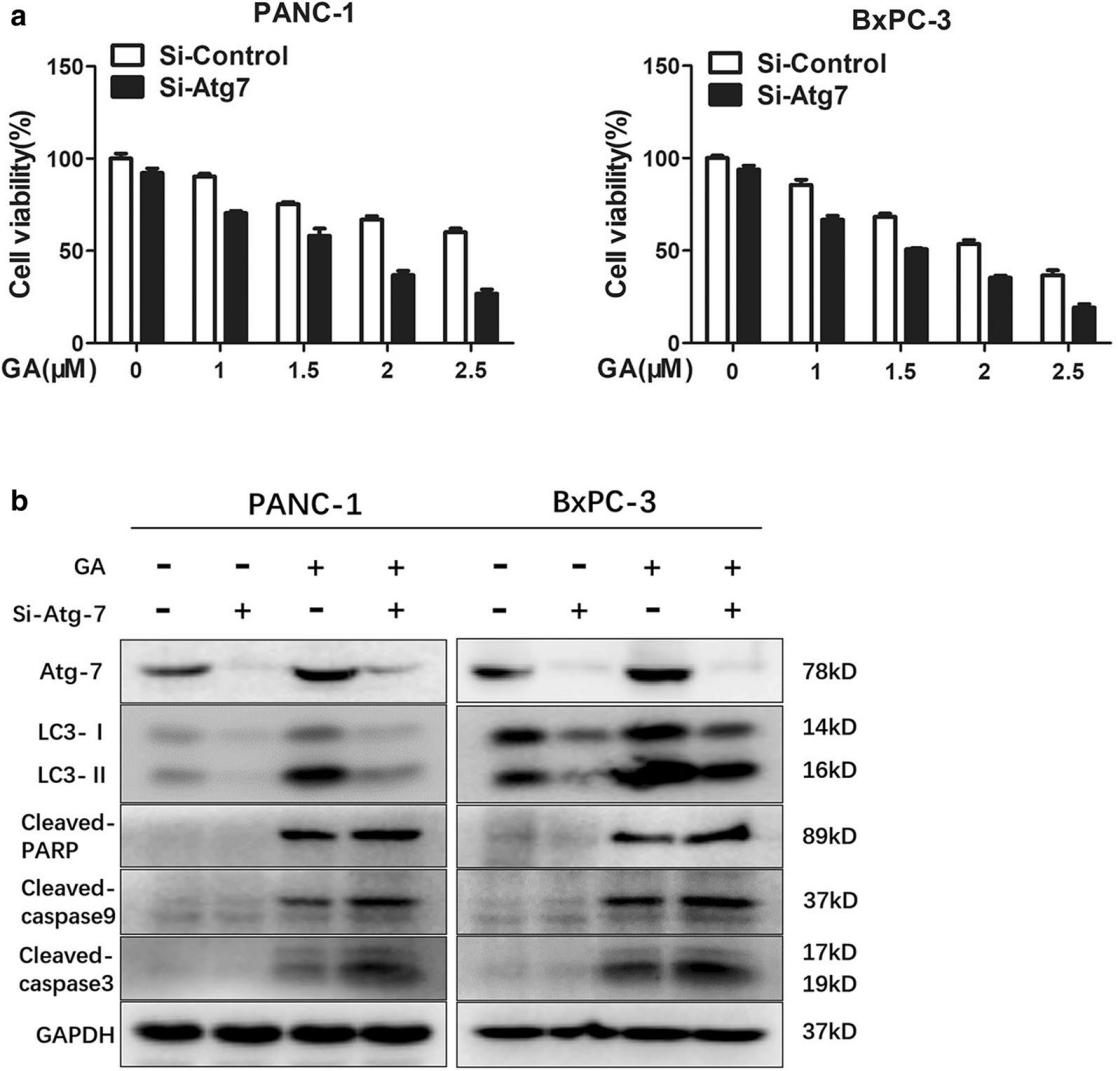
Inhibition of autophagy with knockdown of Atg-7 Si-RNA enhanced gambogic acid (GA) cytotoxicity in cells. PANC-1 and BxPC-3 cells were transiently transfected with Si-Atg-7, cultured for 48 h, and then treated with GA for another 24 h. Cell viability was detected by the MTT assay (**a**). **b** The expression of Atg-7, LC-3, cleaved-PARP, cleaved caspase-3, and cleaved caspase-9 proteins was analyzed by western blot. Data are presented as mean ± SD (n = 3)

To determine whether autophagy is involved in GA-induced apoptosis in pancreatic cancer, cell viability and apoptosis assays were performed. As shown in Fig. 2a, PANC-1 and BxPC-3 cells were pretreated with CQ for 24 h, following which they were incubated with GA at different concentrations for 24 h. The cell viability assay results showed that the cytotoxicity of GA was promoted by CQ. The combination index (shown in Fig. 2b) indicated the synergistic effect between CQ and GA. In addition, the apoptosis assay showed consistent results (Fig. 2c). To further study the combined effect of CQ with GA in pancreatic cancer cells, the expression of the apoptosis-associated proteins cleaved-PARP and cleaved caspase-9 was examined by immunoblot (Fig. 2d). The results showed that CQ promoted the ability of GA to induce the expression of cleaved-PARP and cleaved caspase-9, and identical results were observed in PANC-1 and BxPC-3 cells treated with 3-MA and GA. To further ascertain whether the inhibition of autophagy could promote the cytotoxicity of GA, we inhibited autophagy using Si-RNA to knockdown mRNA levels of *ATG*-*7*, which is the key gene promoting the autophagic process. The results of the cell viability assay and immunoblot showed that knockdown of *ATG*-*7* played a similar role with CQ (Fig. 3a, b). Overall, these findings indicate that the inhibition of autophagy could enhance the pro-apoptotic effect of GA in pancreatic cancer cells, and suggests that GA-induced autophagy plays a cytoprotective role.

### GA induces autophagy by inhibiting the mammalian target of rapamycin (mTOR) pathway

**Fig. 4:**
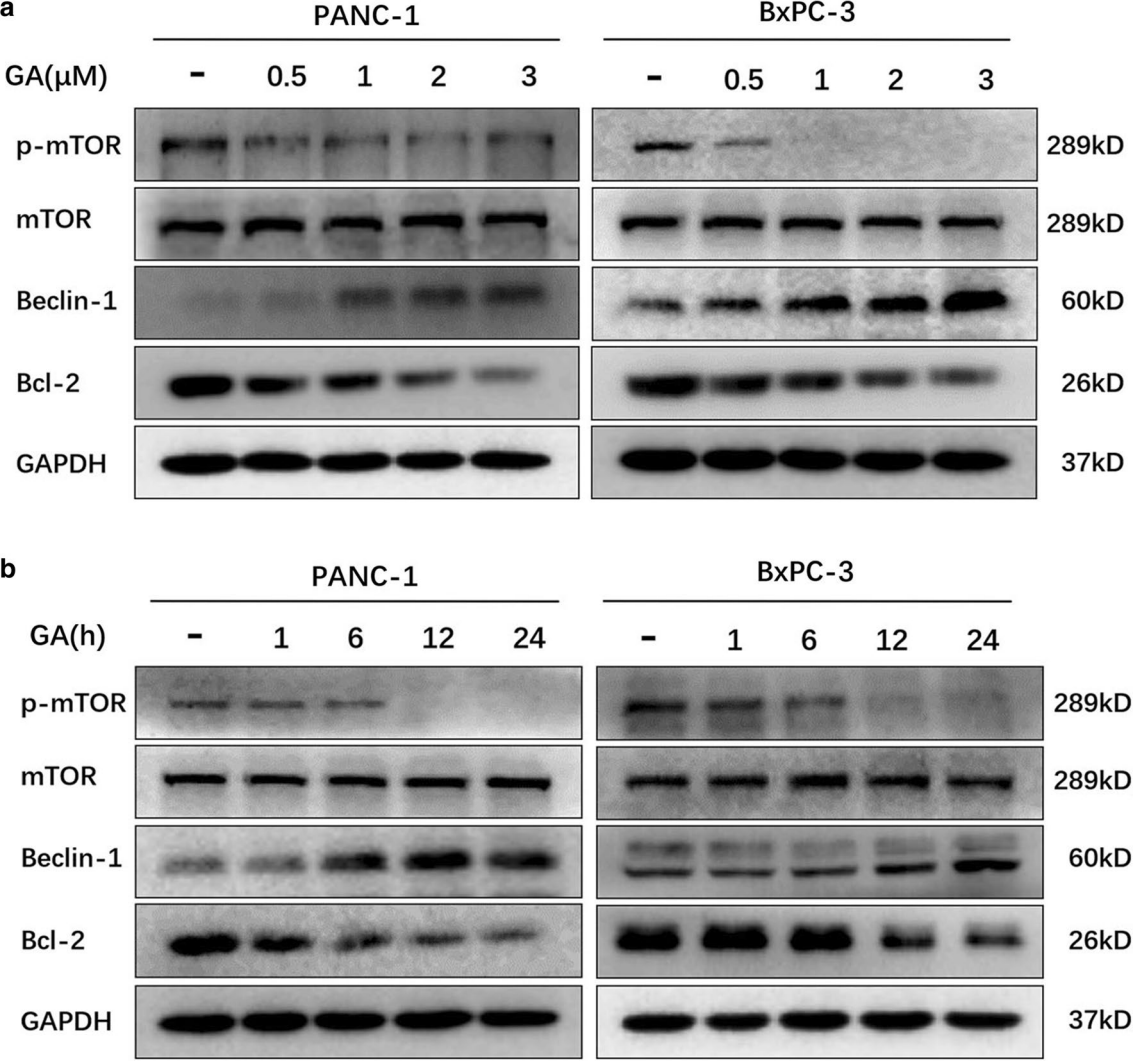
Gambogic acid (GA) inhibited p-mTOR and Bcl-2 expression, and increased Beclin-1 expression in cells. **a** PANC-1 and BxPC-3 cells were either treated with the indicated concentrations of GA for 24 h, or 1 µM GA for the indicated times. **b** The protein expression of p-mTOR, mTOR, Bcl-2, and Beclin-1 was analyzed by western blot

Autophagy can be induced when the AKT/mTOR pathway is inhibited. Our previous study demonstrated that GA inhibited the phosphorylation of AKT in pancreatic cancer cells. Thus, we performed immunoblot analysis to investigate whether GA inhibits the phosphorylation of mTOR. The results showed that GA reduces the expression of p-mTOR (Fig. 4a, b) in a time- and dose-dependent manner. Evidence has shown that inhibition of the mTOR pathway activates the autophagic promoter Beclin-1, and Bcl-2 can deactivate Beclin-1 [16, 18]. Here, we found that GA reduced the expression of Bcl-2 and increased the expression of Beclin-1 in a dose- and time-dependent manner (Fig. 4a, b). These findings suggest that GA could promote autophagy by inhibiting the mTOR pathway.

### GA promotes the generation of ROS through mitochondrial damage and induces autophagy in pancreatic cancer cells

**Fig. 5:**
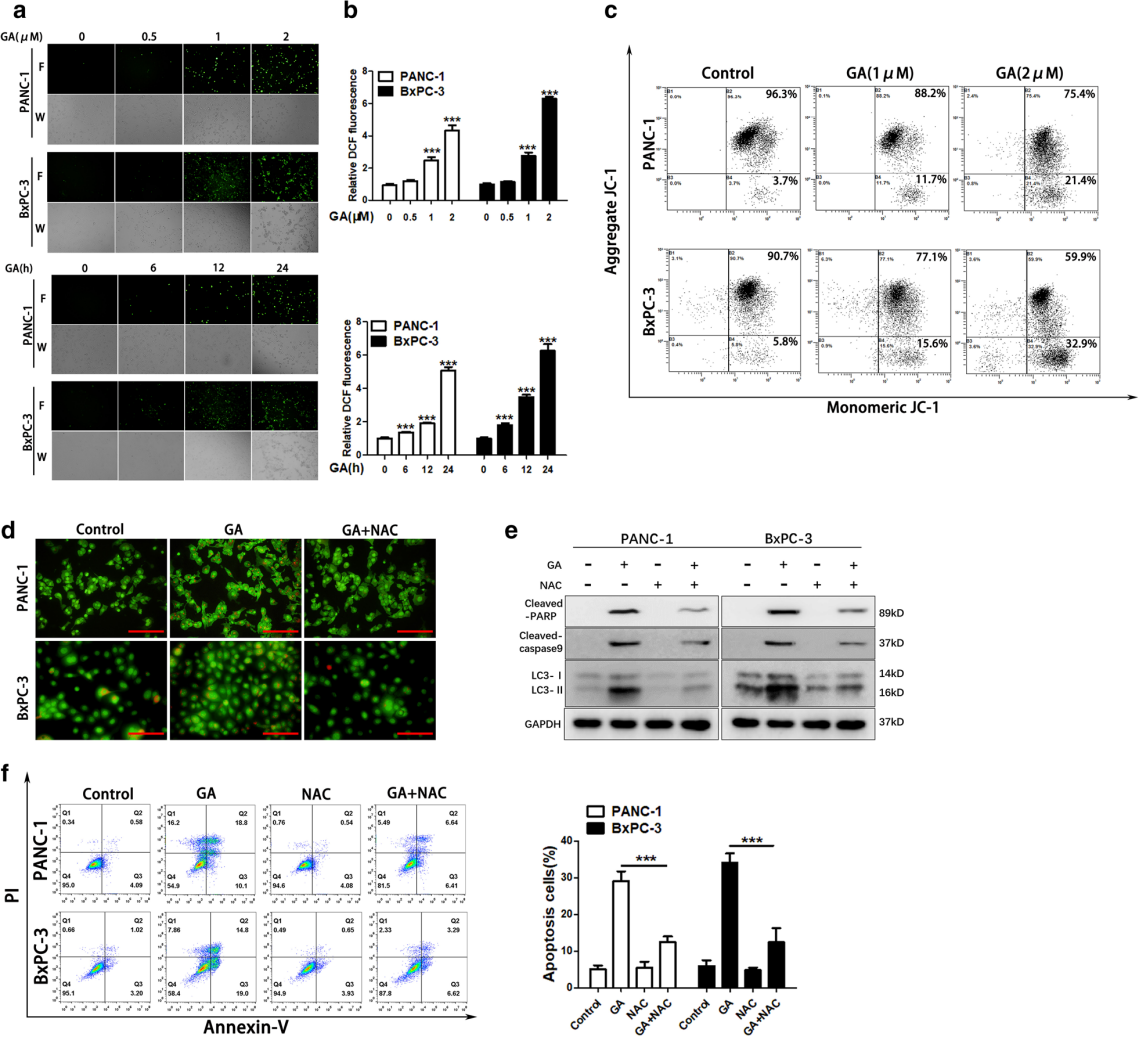
Gambogic acid (GA) promotes ROS generation by damaging mitochondria, and induces pancreatic cancer cell autophagy. **a** PANC-1 and BxPC-3 cells were either treated with the indicated concentrations of GA for 24 h, or treated with GA for the indicated times. The generation of reactive oxygen species (ROS) was detected by CM-H2DCFDA staining and observed under a fluorescence microscopes (×100). **b** ROS production was detected by CM-H2DCFDA staining and ROS levels were measured using a fluorescence microplate. **c** PANC-1 and BxPC-3 cells were treated with 1 µM or 2 µM GA for 24 h, and the mitochondrial membrane potential was measured using the JC-1 assay. **d** PANC-1 and BxPC-3 cells were treated with 1 µM GA in the absence or presence of *N*-acetylcysteine (NAC) (20 mM) for 24 h. The acidic vesicular organelles (AVOs) were detected with acridine staining and observed under a fluorescence microscope. Scale bars indicate 200 µm. **e** PANC-1 and BxPC-3 cells were treated with 2 µM GA in the absence or presence of NAC (20 mM) for 24 h, and the expression of cleaved caspase-9, cleaved-PARP, and LC3 proteins was analyzed with western blot. **f** PANC-1 and BxPC-3 cells were treated with GA (2 µM) for 24 h in the presence or absence of NAC (20 nM). Apoptosis was detected using the Annexin V/PI double stain, and flow cytometry was performed. Data are presented as mean ± SD (n = 3); *** indicates *P *< 0.001

Reactive oxygen species (ROS) regulate autophagy and apoptosis, and previous research has reported that GA induces ROS generation in colon and prostate cancer [17, 22]. To determine whether ROS are involved in GA-induced autophagy in pancreatic cancer cells, we detected intracellular ROS production in PANC-1 and BxPC-3 cells treated with GA. As shown in Fig. 5a, b, GA promoted the accumulation of ROS in a dose- and time-dependent manner in BxPC-3 and PANC-1 cells. The ROS were mostly produced by the mitochondria, and mitochondrial damage accounted for ROS accumulation in normal and tumor cells [23]. We examined the mitochondrial damage in PANC-1 and BxPC-3 cells treated with GA by detecting the change in mitochondrial membrane potential. The results showed that GA significantly reduced the mitochondrial membrane potential, suggesting that GA could promote ROS production by inducing mitochondrial damage (Fig. 5c). To further evaluate whether ROS is a critical factor in the autophagic process, we used a ROS scavenger and mitochondrial enhancer, *N*-acetylcysteine (NAC), to suppress ROS generation. The results showed that NAC reduced the expression of LC3-II and formation of AVOs induced by GA (Fig. 5d, e). Furthermore, NAC attenuated GA-induced apoptosis and the expression of cleaved-PARP and cleaved caspase-9 (Fig. 5e, f). These findings suggest that GA could induce autophagy and apoptosis by promoting the accumulation of ROS in pancreatic cancer cells.

### Inhibition of autophagy enhances ROS production in pancreatic cancer cells treated with GA

**Fig. 6:**
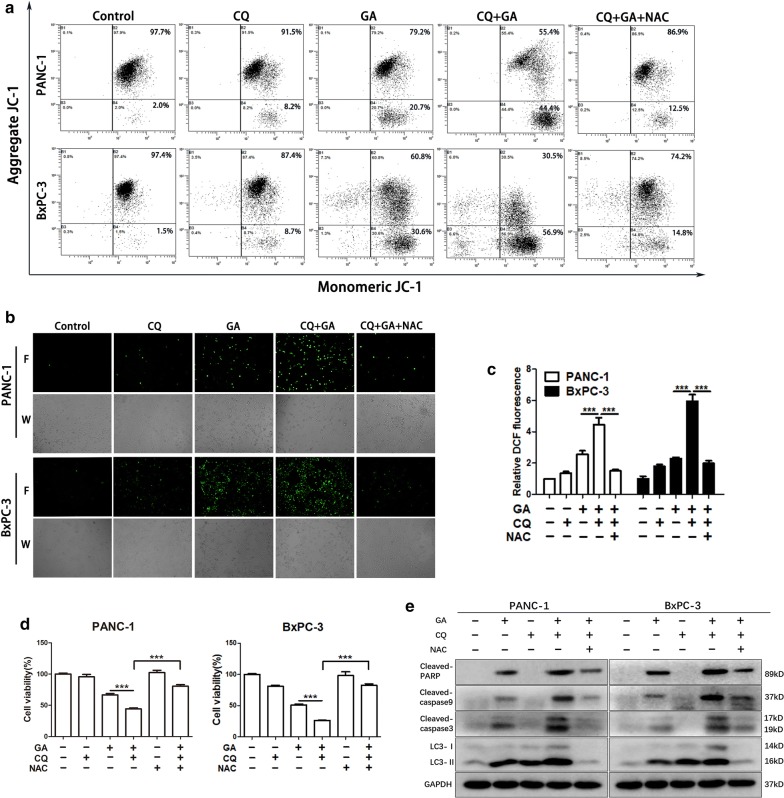
Inhibition of autophagy enhanced ROS production in pancreatic cancer cells treated with gambogic acid (GA). **a** PANC-1 and BxPC-3 cells were pretreated with 40 µM chloroquine (CQ) for 24 h, and then treated with 2 µM GA for another 24 h in the absence or presence of *N*-acetylcysteine (NAC) (20 mM), and the mitochondrial membrane potential was measured using the JC-1 assay. **b**–**d** PANC-1 and BxPC-3 cells were pretreated with 40 µM CQ for 24 h, and then treated with 1 µM GA for another 24 h in the absence or presence of NAC (20 mM). Reactive oxygen species (ROS) production was detected by CM-H2DCFDA staining and observed under a fluorescence microscope (×100) (**b**). ROS production was detected by CM-H2DCFDA staining and ROS levels were measured using a fluorescence microplate (**c**). Cell viability was detected by the MTT assay (**d**). **e** PANC-1 and BxPC-3 cells were pretreated with 40 µM CQ for 24 h, and then treated with 2 µM GA for another 24 h in the absence or presence of NAC (20 mM), and the expression of cleaved caspase-9, cleaved-PARP, cleaved caspase-3, and LC3 proteins was analyzed with western blot. Data are presented as mean ± SD (n = 3); *** indicates *P *< 0.001

Mitochondrial damage produces large amounts of ROS, which induce autophagy in healthy cells and remove damaged mitochondria and peroxides to protect-themselves [24], Thus, the inhibition of autophagy causes the accumulation of ROS under the conditions of mitochondrial damage. The aforementioned results demonstrate that GA induces cell protective autophagy in pancreatic cancer cells, and the inhibition of autophagy relieved its protective function. To further demonstrate whether the inhibition of autophagy causes ROS accumulation under GA treatment, we detected the mitochondrial membrane potential and ROS levels in pancreatic cancer cells. As shown in Fig. 6a, GA significantly reduced the mitochondrial membrane potential in PANC-1 and BxPC-3 cells, which was further reduced under CQ treatment. However, the presence of NAC ameliorated these effects. The ROS levels were evidently higher in the combination treatment group, and NAC significantly reduced ROS production (Fig. 6b, c). These results suggest that the blocking of autophagy could impair the clearance of damaged mitochondria from pancreatic cancer cells, contribute to the excessive accumulation of ROS, and eventually lead to apoptosis. Consistently, as shown in Fig. 4d, the expression of the apoptosis-related proteins, cleaved-PARP, cleaved caspase-3, and cleaved caspase-9 was significantly elevated when cells were co-treated with CQ and GA. In contrast, when the ROS were eliminated by NAC, the expression of the aforementioned proteins was reduced, and cell viability showed a similar trend. As the activation of autophagy could eliminate damaged mitochondria and reduce ROS production, we investigated whether the pre-activation of autophagy with rapamycin in pancreatic cancer cells would reduce GA-induced ROS production. We found that the activation of autophagy prior to treatment with GA reduced ROS levels (Additional file 1). Furthermore, this activation reduced the cytotoxicity of GA (Additional file 2).

### GA and CQ act synergistically against pancreatic cancer in vivo

**Fig. 7:**
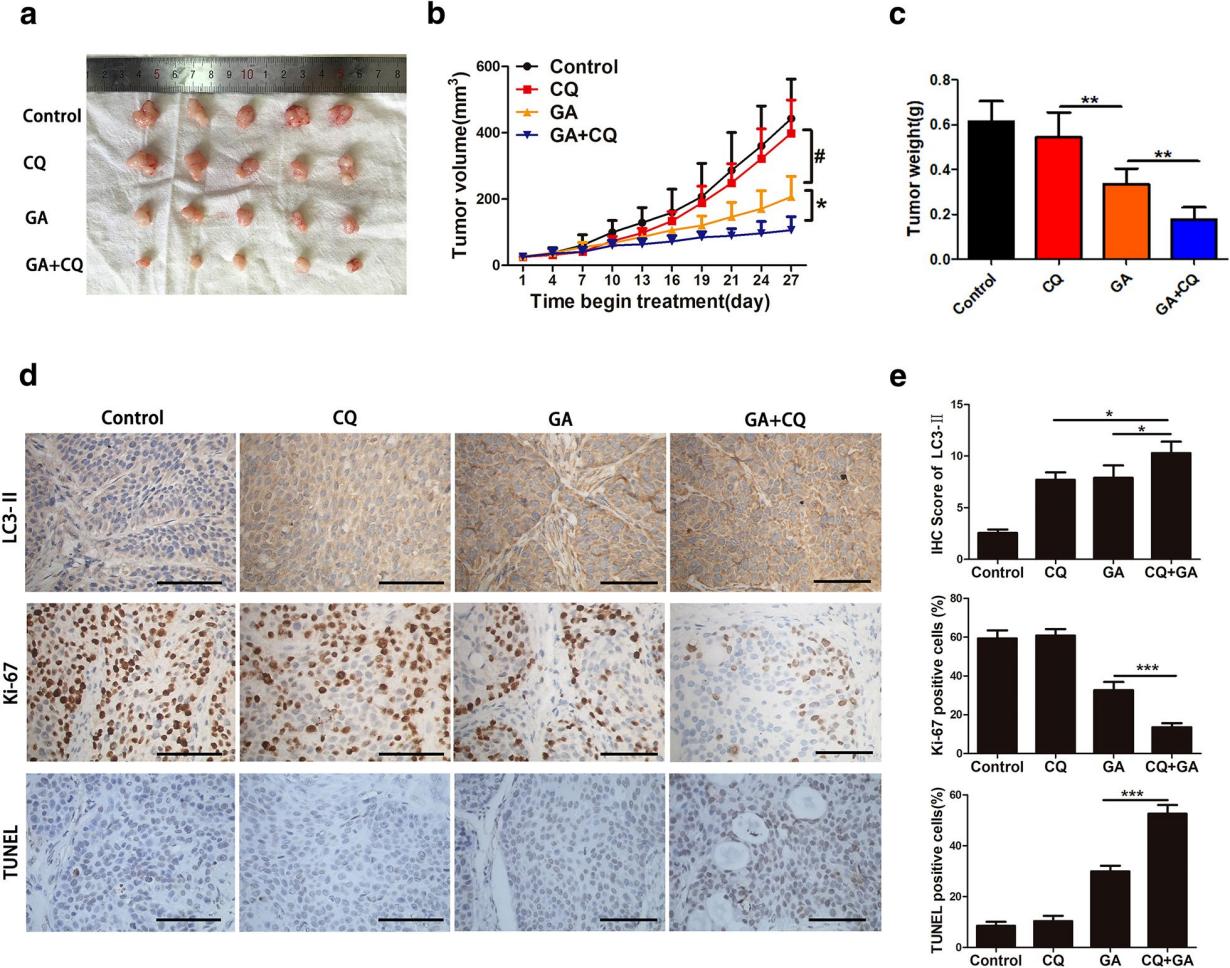
Gambogic acid (GA) and chloroquine (CQ) act synergistically against a pancreatic cancer xenograft tumor model. Xenograft tumor model was created with BxPC-3 cells in nude mice. **a** Xenograft tumors of four groups. **b** Tumor volume of four groups was measured every 3 days. * indicates the CQ + GA group compared to the GA group, *P *< 0.05; ^#^ indicates the GA group compared to the CQ group, *P *< 0.05. **c** Tumor weight. **d** Immunohistochemical (IHC) analysis of LC3-II, Ki-67, and TUNEL in xenograft tumor specimens. Scale bars indicate 200 µm. **e** Quantification of IHC staining of LC3-II, Ki-67, and terminal deoxynucleotidyl transferase dUTP nick end labeling (TUNEL). Data are presented as mean ± SD (n = 5), * indicates *P *< 0.05; ** indicates *P *< 0.01; *** indicates *P *< 0.001

To further validate the efficacy of combined treatment with CQ and GA against pancreatic cancer cells in vivo, BxPC-3 cells were used to create a xenograft tumor model in nude mice. Tumor-bearing mice were randomly divided into four groups, which were respectively treated with saline, CQ, GA, and CQ + GA. The treatment duration in all groups was 27 days. Xenograft tumors in the four groups are shown in Fig. 7a. As depicted in Fig. 7b, CQ treatment had little effect on tumor growth in comparison to the control. As expected, treatment with GA or GA combined with CQ significantly inhibited tumor growth, and the combined treatment had superior efficacy to the GA treatment alone. Tumor weight was also measured in the four groups, and the results were consistent with those of tumor volume (Fig. 7c). Further IHC experiments were performed to explore the expression of Ki-67 and LC3-II in xenograft tumor tissues, and the TUNEL assay was used to detect apoptosis. As shown in Fig. 7d, Ki-67 staining confirmed a significant reduction in proliferation in the GA and combined treatment groups, with the latter group showing less proliferation than the former. In contrast, the results of the TUNEL assay showed that the combination group showed the highest levels of apoptosis in comparison to other groups. The CQ, GA, and combined treatments all increased LC3-II staining, which was consistent with the results observed in vitro. These findings all corroborated the in vitro results and demonstrated the synergistic antitumor effects of combining GA with the autophagy inhibitor, CQ, in pancreatic cancer. The combination of GA and CQ could be a potential treatment regimen for patients with pancreatic cancer.

## Discussion

In the present study, we found that GA induced autophagy and apoptosis in pancreatic cancer cells. Furthermore, GA-induced autophagy was able to protect pancreatic cancer cells from apoptosis, which was associated with ROS scavenging and mitochondrial damage. Moreover, the inhibition of autophagy with CQ eliminated the autophagy-induced cell survival effect and increased the cytotoxicity of GA in vitro and in vivo.

Autophagy is a very common phenomenon and a highly conserved cellular process that plays an important role in maintaining homeostasis in cells. It plays ambiguous roles in various cancers and the mechanisms remain unknown [5, 25, 26]. High expression of the autophagy-associated genes, *LC3* and *Beclin*-*1*, are reportedly associated with a poor prognosis in various cancer patients [27–29]. However, studies also demonstrate that autophagy induces cell death and acts as a tumor suppressor [30, 31]. In pancreatic cancer, increasing evidence suggests that autophagy plays a cytoprotective role under conditions of cellular stress and chemotherapy [32–34]. The cytoprotective roles of autophagy confer chemotherapeutic resistance. Various types of chemotherapeutics could reportedly induce autophagy in pancreatic cancer and lead to chemoresistance [14, 35]. We found that the inhibition of autophagy in pancreatic cancer cells augmented the cytotoxicity of GA in vitro and in vivo. Moreover, we also found that the activation of autophagy with rapamycin at low concentrations could promote pancreatic cancer cell survival under GA treatment. These findings indicate that GA-induced autophagy is a cytoprotective autophagy. Degenhardt et al.’s study demonstrated that autophagy promoted tumor cell survival by preventing apoptosis and death [36]. Marchand et al. found that autophagy induced by the inhibition of GSK3 promotes pancreatic cancer cell survival [32].

The mechanism by which autophagy is induced has been widely reported, and inhibition of the AKT/mTOR, ROS/AMPK, and Bcl-2/Beclin-1 signaling pathways are known to induce autophagy in cancer cells [24, 37, 38]. Our previous study showed that GA inhibits the phosphorylation of AKT in pancreatic cancer cells [18]. Accumulated evidence demonstrates that inhibition of AKT/mTOR signaling pathway activates Beclin-1 which is the key regulator of autophagy [4, 39]. In this study, we found that GA inhibited the phosphorylation of mTOR in a dose and time dependent manner, and the expression of beclin-1 also increased, suggesting that GA could activate beclin-1 through inhibiting AKT/mTOR signaling pathway. AKT/mTOR signaling pathway also plays an important role in cell growth, studies have confirmed that inhibition of it induced cell apoptosis [40], which indicated that GA-induced cell apoptosis also was partly contributed to the inhibition of AKT/mTOR signaling pathway. Meanwhile, GA downregulated the expression of P62, and promoted the autophagic flux and the generation of AVOs in pancreatic cancer cells, which all suggested that autophagy was induced by GA.

As a regulator of PCD (programmed cell death), Bcl-2 is also an important factor in the regulation of autophagy. It inhibits autophagy by binding to and impeding Beclin-1, which plays a central role in promoting autophagy [41]. Our study revealed that GA suppresses the expression of Bcl-2, and increases the expression of Beclin-1 to activate autophagy. Moreover, Bcl-2 is known as a tumor suppressor, which inhibits apoptosis and promotes cell survival. Thus, the inhibition of Bcl-2 could also explain why GA is able to induce apoptosis [42]. An alternative way to induce autophagy is via ROS, which could activate AMPK and lead to the inhibition of the mTOR signaling pathway. The ROS can also transcriptionally augment the expression of P62 through KEAP1/NRF2 activation [31, 33]. Our results showed that ROS levels were significantly elevated in pancreatic cancer cells under GA treatment. Furthermore, we demonstrated that ROS was required for GA-induced autophagy.

The ROS are chemically reactive species that can be produced by mitochondria during the process of oxidative phosphorylation in cells. They can induce both apoptosis and autophagy [43]. Studies have demonstrated that oxidative stress produces ROS by damaging the mitochondria. Conversely, ROS-induced autophagy can scavenge the damaged mitochondria and sustain cellular homeostasis. Moreover, the accumulation of ROS can occur if the stimulation of stress is sustained, and can eventually lead to apoptosis and death [44–46]. Therefore, one possible method by which the accumulation of ROS can be induced in cancer therapy is through the inhibition of autophagy. However, the inhibition of autophagy alone as a therapeutic strategy for cancer would not necessarily have favorable therapeutic effects [20]. Our results showed that the inhibition of autophagy with CQ in pancreatic cancer cells had inferior antitumor effects in vitro and in vivo. We also found that the ROS levels in pancreatic cancer cells treated with CQ showed only a limited increase, whereas the mitochondrial membrane potentials remained almost the same as those of untreated pancreatic cancer cells. These findings suggest that the inhibition of autophagy alone may not sufficiently increase ROS levels to induce apoptosis. In contrast, when we co-treated pancreatic cancer cells with CQ and GA, the ROS levels were significantly increased and mitochondrial membrane potentials were significantly reduced. Moreover, when we co-treated pancreatic cancer cells with rapamycin and GA, the GA-induced ROS production and apoptosis were both reduced. These findings indicate that the activation of autophagy could reduce ROS production by scavenging the damaged mitochondria, which effectively protects the cells from apoptosis. Furthermore, the inhibition of autophagy could also inhibit ROS scavenging and thereby lead to ROS accumulation, and eventually apoptosis. Previous reports have also shown that cellular senescence, apoptosis, and autophagy are interconnected. The study of Squillaro et al. demonstrated that impaired autophagy could promote senescence and apoptosis in mesenchymal stem cells (MSCs) [47]. Like autophagy, senescence has defined roles in tumor suppression and tumor promotion. However, the regulation of interactions between the two processes still requires further research [48]. The present study showed that GA could simultaneously induce autophagy and apoptosis in pancreatic cancer cells, suggesting that GA-induced autophagy might share a relationship with senescence. However, this association requires further elucidation.

Cell protective autophagy is reportedly induced by antitumor agents, and this can lead to the development of resistance to cancer therapy [49–51]. Therefore, the inhibition of protective autophagy may enhance drug resistance. Our previous study demonstrated that GA induced apoptosis by directly activating the caspase pathway [18]. Intracellular accumulation of ROS can also induce apoptosis by activating the caspase pathway [24, 52]. The results of the present study showed that GA induced the expression of apoptosis-associated proteins by increasing ROS levels, and NAC was able to inhibit GA-induced apoptosis. These findings suggest that GA could directly and indirectly activate the caspase pathway. Our data also showed that GA induced protective autophagy, which limited its cytotoxicity. Furthermore, the inhibition of autophagy increased the GA-induced ROS production and led to apoptosis, thereby alleviating GA-induced cytoprotective autophagy and mitigating GA-resistance. Most remarkably, the combined treatment of CQ and GA showed the greatest antitumor effect in vivo. The above data all indicate that the inhibition of autophagy could effectively promote the cytotoxicity of GA in pancreatic cancer.

## Conclusions

Collectively, our findings demonstrate that GA induces cytoprotective autophagy in pancreatic cancer cells, and the inhibition of autophagy augments the cytotoxic effects of GA against pancreatic cancer. These results provide a potential and promising combination treatment of GA and CQ for pancreatic cancer. However, the combined therapeutic approach needs to be validated by follow-up clinical studies.

## Additional files

**Additional file 1.** Rapamycin reduced GA-induced reactive oxygen species (ROS) production in pancreatic cancer cells. PANC-1 and BxPC-3 cells were pretreated with 200 nM rapamycin for 24 h, and then treated with 1 µM GA for another 24 h. The generation of reactive oxygen species (ROS) was detected by CM-H2DCFDA staining and observed under a fluorescence microscopes (100×). ROS production was detected by CM-H2DCFDA staining and ROS levels were measured using a fluorescence microplate. Data are presented as mean ± SD (n = 3); ** indicates *P* < 0.01, *** indicates *P *< 0.001.

**Additional file 2.** Rapamycin reduced the cytotoxicity of GA in pancreatic cancer cells. PANC-1 and BxPC-3 cells were pretreated with either 200 nM or 500 nM rapamycin for 24 h, and then treated with 1 µM GA for another 24 h. Cell viability was detected by the MTT assay. Data are presented as mean ± SD (n = 3); ** indicates *P *< 0.01, *** indicates *P *< 0.001.

## Abbreviations

3MA: 3-methyladenine
AKT: protein kinase B
AMPK: AMP-activated protein kinase
AVO: acidic vesicular organelles
Baf-A1: bafilomycin A1
CI: combination index
CQ: chloroquine
DAB: diaminobenzidine
DMEM: Dulbecco’s modified Eagle’s medium
DMSO: dimethyl sulfoxide
GA: gambogic acid
IHC: immunohistochemical
mTOR: mammalian target of rapamycin
MTT: 3-(4,5-dimethylthiazol-2-yl)-2,5-diphenyl tetrazolium bromide
NAC: *N*-acetylcysteine
PBS: phosphate-buffered saline
PCD: programmed cell death
PI: phosphoinositide
PMSF: phenylmethylsulfonyl fluoride
PVDF: polyvinylidene difluoride
RIPA: radioimmunoprecipitation assay
ROS: reactive oxygen species
SDS-PAGE: sodium dodecyl sulfate-polyacrylamide gel electrophoresis
TUNEL: terminal deoxynucleotidyl transferase dUTP nick end labeling

## References

[1] Siegel RL, Miller KD, Jemal A (2018). Cancer statistics, 2018. CA Cancer J Clin.

[2] Paulson AS, Tran Cao HS, Tempero MA, Lowy AM (2013). Therapeutic advances in pancreatic cancer. Gastroenterology.

[3] Neoptolemos JP (2011). Adjuvant treatment of pancreatic cancer. Eur J Cancer.

[4] Hale AN, Ledbetter DJ, Gawriluk TR, Rucker EB (2013). Autophagy: regulation and role in development. Autophagy.

[5] Ravikumar B, Sarkar S, Davies JE, Futter M, Garcia-Arencibia M, Green-Thompson ZW, Jimenez-Sanchez M, Korolchuk VI, Lichtenberg M, Luo S (2010). Regulation of mammalian autophagy in physiology and pathophysiology. Physiol Rev.

[6] Mizushima N, Levine B, Cuervo AM, Klionsky DJ (2008). Autophagy fights disease through cellular self-digestion. Nature.

[7] Mathew R, Karantza-Wadsworth V, White E (2007). Role of autophagy in cancer. Nat Rev Cancer.

[8] Neesse A, Michl P, Frese KK, Feig C, Cook N, Jacobetz MA, Lolkema MP, Buchholz M, Olive KP, Gress TM (2011). Stromal biology and therapy in pancreatic cancer. Gut.

[9] Rosenfeldt MT, O’Prey J, Morton JP, Nixon C, MacKay G, Mrowinska A, Au A, Rai TS, Zheng L, Ridgway R (2013). p53 status determines the role of autophagy in pancreatic tumour development. Nature.

[10] Endo S, Nakata K, Ohuchida K, Takesue S, Nakayama H, Abe T, Koikawa K, Okumura T, Sada M, Horioka K (2017). Autophagy is required for activation of pancreatic stellate cells, associated with pancreatic cancer progression and promotes growth of pancreatic tumors in mice. Gastroenterology.

[11] Cebrian MJ, Bauden M, Andersson R, Holdenrieder S, Ansari D (2016). Paradoxical role of HMGB1 in pancreatic cancer: tumor suppressor or tumor promoter?. Anticancer Res.

[12] Fiorini C, Cordani M, Gotte G, Picone D, Donadelli M (2015). Onconase induces autophagy sensitizing pancreatic cancer cells to gemcitabine and activates Akt/mTOR pathway in a ROS-dependent manner. Biochim Biophys Acta.

[13] Xu XD, Zhao Y, Zhang M, He RZ, Shi XH, Guo XJ, Shi CJ, Peng F, Wang M, Shen M (2017). Inhibition of autophagy by deguelin sensitizes pancreatic cancer cells to doxorubicin. Int J Mol Sci.

[14] Yang MC, Wang HC, Hou YC, Tung HL, Chiu TJ, Shan YS (2015). Blockade of autophagy reduces pancreatic cancer stem cell activity and potentiates the tumoricidal effect of gemcitabine. Mol Cancer.

[15] Abdel-Aziz AK, Abdel-Naim AB, Shouman S, Minucci S, Elgendy M (2017). From resistance to sensitivity: insights and implications of biphasic modulation of autophagy by sunitinib. Front Pharmacol.

[16] Kashyap D, Mondal R, Tuli HS, Kumar G, Sharma AK (2016). Molecular targets of gambogic acid in cancer: recent trends and advancements. Tumour Biol.

[17] Ishaq M, Khan MA, Sharma K, Sharma G, Dutta RK, Majumdar S (2014). Gambogic acid induced oxidative stress dependent caspase activation regulates both apoptosis and autophagy by targeting various key molecules (NF-kappaB, Beclin-1, p62 and NBR1) in human bladder cancer cells. Biochim Biophys Acta.

[18] Xia G, Wang H, Song Z, Meng Q, Huang X, Huang X (2017). Gambogic acid sensitizes gemcitabine efficacy in pancreatic cancer by reducing the expression of ribonucleotide reductase subunit-M2 (RRM2). J Exp Clin Cancer Res.

[19] Foggetti G, Ottaggio L, Russo D, Monti P, Degan P, Fronza G, Menichini P (2017). Gambogic acid counteracts mutant p53 stability by inducing autophagy. Biochim Biophys Acta.

[20] Li X, Zhu F, Jiang J, Sun C, Zhong Q, Shen M, Wang X, Tian R, Shi C, Xu M (2016). Simultaneous inhibition of the ubiquitin-proteasome system and autophagy enhances apoptosis induced by ER stress aggravators in human pancreatic cancer cells. Autophagy.

[21] Vena F, Li Causi E, Rodriguez-Justo M, Goodstal S, Hagemann T, Hartley JA, Hochhauser D (2015). The MEK1/2 inhibitor pimasertib enhances gemcitabine efficacy in pancreatic cancer models by altering ribonucleotide reductase subunit-1 (RRM1). Clin Cancer Res.

[22] Filomeni G, De Zio D, Cecconi F (2015). Oxidative stress and autophagy: the clash between damage and metabolic needs. Cell Death Differ.

[23] Lee J, Giordano S, Zhang J (2012). Autophagy, mitochondria and oxidative stress: cross-talk and redox signalling. Biochem J.

[24] Poillet-Perez L, Despouy G, Delage-Mourroux R, Boyer-Guittaut M (2015). Interplay between ROS and autophagy in cancer cells, from tumor initiation to cancer therapy. Redox Biol.

[25] Liang XH, Jackson S, Seaman M, Brown K, Kempkes B, Hibshoosh H, Levine B (1999). Induction of autophagy and inhibition of tumorigenesis by beclin 1. Nature.

[26] Yue Z, Jin S, Yang C, Levine AJ, Heintz N (2003). Beclin 1, an autophagy gene essential for early embryonic development, is a haploinsufficient tumor suppressor. Proc Natl Acad Sci USA.

[27] Fujii S, Mitsunaga S, Yamazaki M, Hasebe T, Ishii G, Kojima M, Kinoshita T, Ueno T, Esumi H, Ochiai A (2008). Autophagy is activated in pancreatic cancer cells and correlates with poor patient outcome. Cancer Sci.

[28] Toton E, Lisiak N, Sawicka P, Rybczynska M (2014). Beclin-1 and its role as a target for anticancer therapy. J Physiol Pharmacol.

[29] He Y, Zhao X, Subahan NR, Fan L, Gao J, Chen H (2014). The prognostic value of autophagy-related markers beclin-1 and microtubule-associated protein light chain 3B in cancers: a systematic review and meta-analysis. Tumour Biol.

[30] Farrall AL, Whitelaw ML (2009). The HIF1alpha-inducible pro-cell death gene BNIP3 is a novel target of SIM2s repression through cross-talk on the hypoxia response element. Oncogene.

[31] Navarro-Yepes J, Burns M, Anandhan A, Khalimonchuk O, del Razo LM, Quintanilla-Vega B, Pappa A, Panayiotidis MI, Franco R (2014). Oxidative stress, redox signaling, and autophagy: cell death versus survival. Antioxid Redox Signal.

[32] Marchand B, Arsenault D, Raymond-Fleury A, Boisvert FM, Boucher MJ (2015). Glycogen synthase kinase-3 (GSK3) inhibition induces prosurvival autophagic signals in human pancreatic cancer cells. J Biol Chem.

[33] Hambright HG, Ghosh R (2017). Autophagy: in the cROSshairs of cancer. Biochem Pharmacol.

[34] Petibone DM, Majeed W, Casciano DA (2017). Autophagy function and its relationship to pathology, clinical applications, drug metabolism and toxicity. J Appl Toxicol.

[35] Li X, Roife D, Kang Y, Dai B, Pratt M, Fleming JB (2016). Extracellular lumican augments cytotoxicity of chemotherapy in pancreatic ductal adenocarcinoma cells via autophagy inhibition. Oncogene.

[36] Mooney JJ, Cole JO, Schatzberg AF, Gerson B, Schildkraut JJ (1985). Pretreatment urinary MHPG levels as predictors of antidepressant responses to alprazolam. Am J Psychiatry.

[37] Kim J, Kundu M, Viollet B, Guan KL (2011). AMPK and mTOR regulate autophagy through direct phosphorylation of Ulk1. Nat Cell Biol.

[38] Bellot G, Garcia-Medina R, Gounon P, Chiche J, Roux D, Pouyssegur J, Mazure NM (2009). Hypoxia-induced autophagy is mediated through hypoxia-inducible factor induction of BNIP3 and BNIP3L via their BH3 domains. Mol Cell Biol.

[39] Duran A, Amanchy R, Linares JF, Joshi J, Abu-Baker S, Porollo A, Hansen M, Moscat J, Diaz-Meco MT (2011). p62 is a key regulator of nutrient sensing in the mTORC1 pathway. Mol Cell.

[40] Laplante M, Sabatini DM (2012). mTOR signaling in growth control and disease. Cell.

[41] Zhou F, Yang Y, Xing D (2011). Bcl-2 and Bcl-xL play important roles in the crosstalk between autophagy and apoptosis. FEBS J.

[42] Zhao L, Guo QL, You QD, Wu ZQ, Gu HY (2004). Gambogic acid induces apoptosis and regulates expressions of Bax and Bcl-2 protein in human gastric carcinoma MGC-803 cells. Biol Pharm Bull.

[43] St-Pierre J, Buckingham JA, Roebuck SJ, Brand MD (2002). Topology of superoxide production from different sites in the mitochondrial electron transport chain. J Biol Chem.

[44] Morselli E, Galluzzi L, Kepp O, Marino G, Michaud M, Vitale I, Maiuri MC, Kroemer G (2011). Oncosuppressive functions of autophagy. Antioxid Redox Signal.

[45] Matsuda N, Sato S, Shiba K, Okatsu K, Saisho K, Gautier CA, Sou YS, Saiki S, Kawajiri S, Sato F (2010). PINK1 stabilized by mitochondrial depolarization recruits Parkin to damaged mitochondria and activates latent Parkin for mitophagy. J Cell Biol.

[46] Weinberg F, Hamanaka R, Wheaton WW, Weinberg S, Joseph J, Lopez M, Kalyanaraman B, Mutlu GM, Budinger GR, Chandel NS (2010). Mitochondrial metabolism and ROS generation are essential for Kras-mediated tumorigenicity. Proc Natl Acad Sci USA.

[47] Squillaro T, Antonucci I, Alessio N, Esposito A, Cipollaro M, Melone MAB, Peluso G, Stuppia L, Galderisi U (2017). Impact of lysosomal storage disorders on biology of mesenchymal stem cells: evidences from in vitro silencing of glucocerebrosidase (GBA) and alpha-galactosidase A (GLA) enzymes. J Cell Physiol.

[48] Ruhland MK, Coussens LM, Stewart SA (2016). Senescence and cancer: an evolving inflammatory paradox. Biochim Biophys Acta.

[49] Dielschneider RF, Henson ES, Gibson SB (2017). Lysosomes as oxidative targets for cancer therapy. Oxid Med Cell Longev.

[50] Galadari S, Rahman A, Pallichankandy S, Thayyullathil F (2017). Reactive oxygen species and cancer paradox: to promote or to suppress?. Free Radic Biol Med.

[51] Lei Y, Zhang D, Yu J, Dong H, Zhang J, Yang S (2017). Targeting autophagy in cancer stem cells as an anticancer therapy. Cancer Lett.

[52] Zou Z, Chang H, Li H, Wang S (2017). Induction of reactive oxygen species: an emerging approach for cancer therapy. Apoptosis.

