# Supplementary figures and images for: Gambogic acid induces autophagy and combines synergistically with chloroquine to suppress pancreatic cancer by increasing the accumulation of reactive oxygen species
Source: Cancer Cell Int. 2019 Jan 5;19:7. doi: 10.1186/s12935-018-0705-x (PMC6321668; doi:10.1186/s12935-018-0705-x)

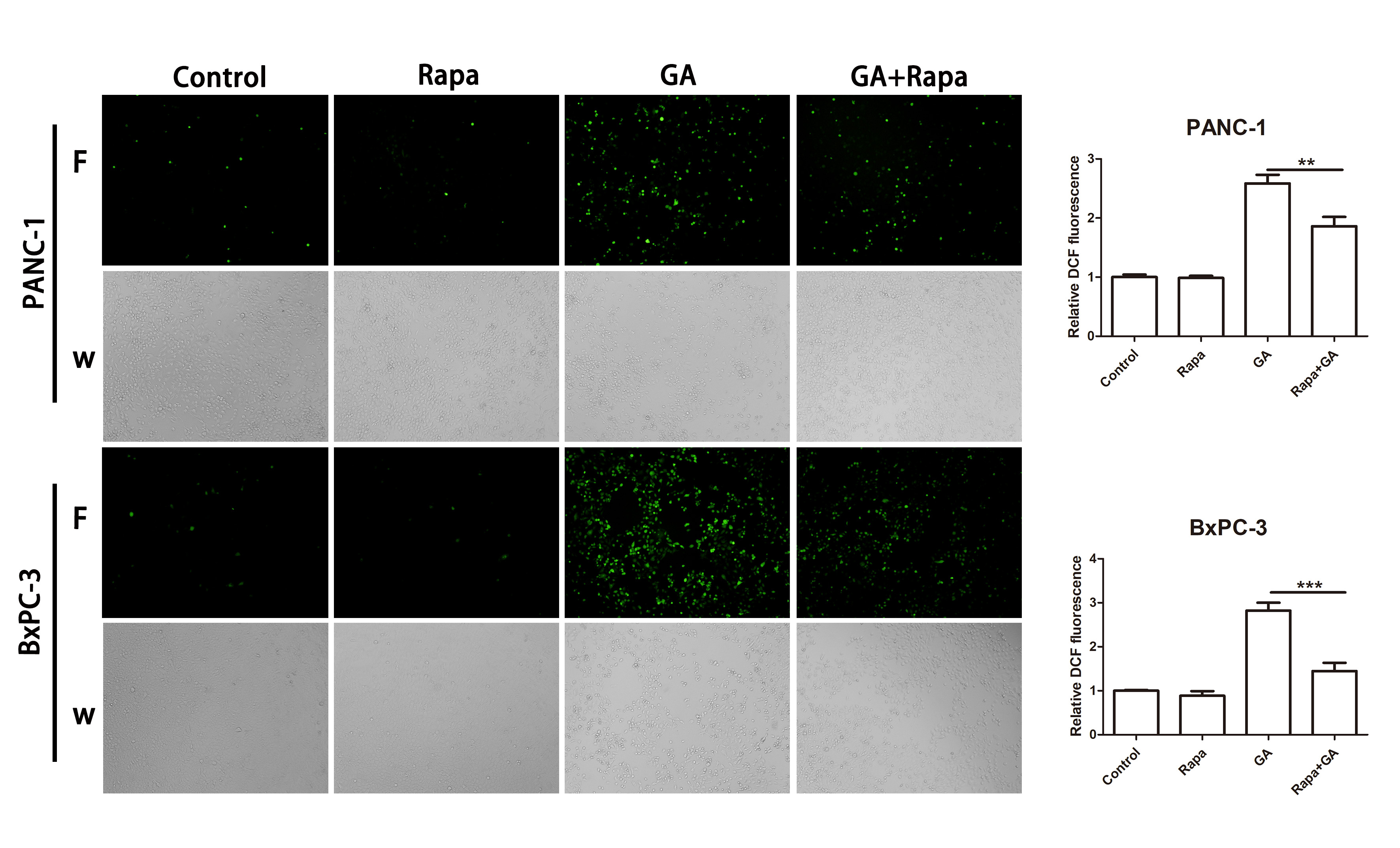

Supplement: Supplementary file 1 — Additional file 1. Rapamycin reduced GA-induced reactive oxygen species (ROS) production in pancreatic cancer cells. PANC-1 and BxPC-3 cells were pretreated with 200 nM rapamycin for 24 h, and then treated with 1 µM GA for another 24 h. The generation of reactive oxygen species (ROS) was detected by CM-H2DCFDA staining and observed under a fluorescence microscopes (100×). ROS production was detected by CM-H2DCFDA staining and ROS levels were measured using a fluorescence microplate. Data are presented as mean ± SD (n = 3); ** indicates P < 0.01, *** indicates P < 0.001. [file 12935_2018_705_MOESM1_ESM.jpg]

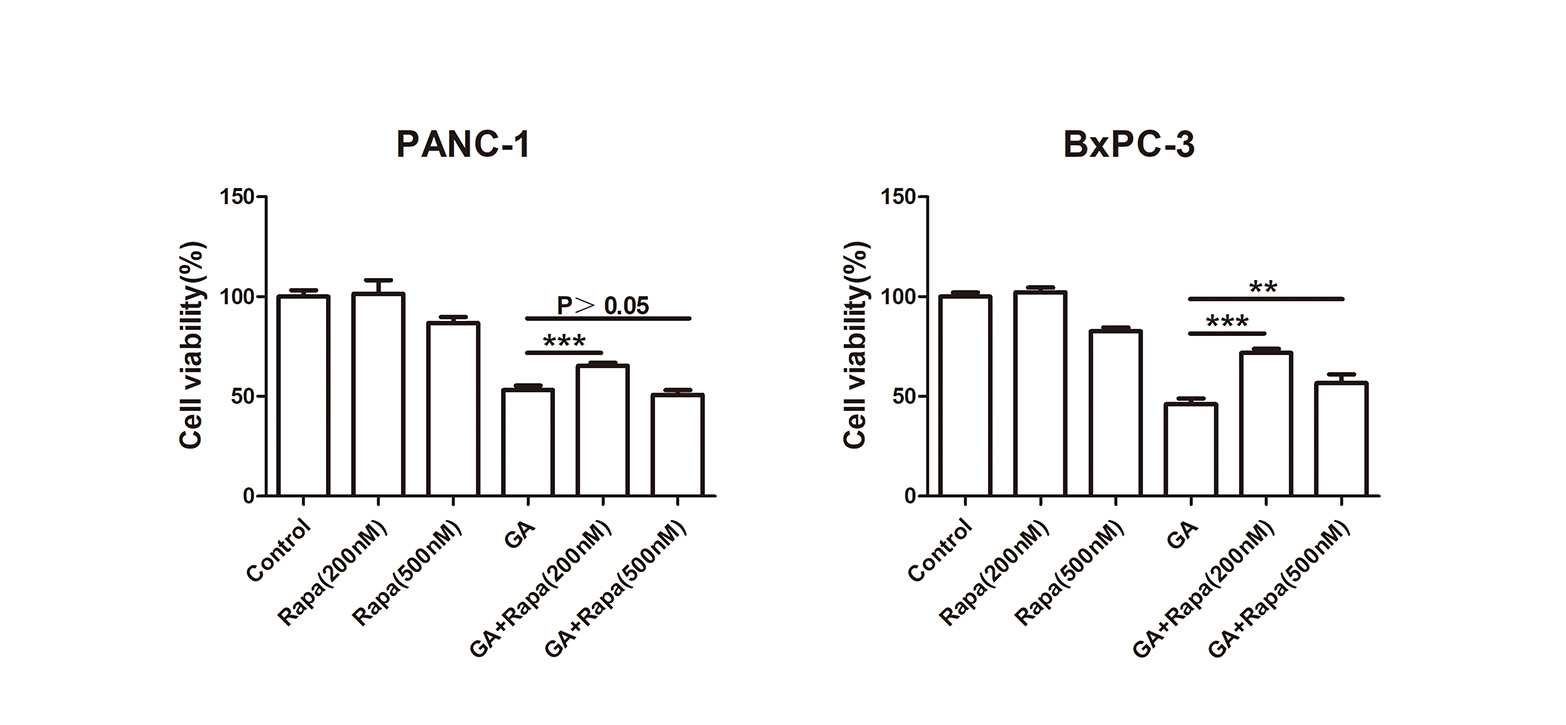

Supplement: Supplementary file 2 — Additional file 2. Rapamycin reduced the cytotoxicity of GA in pancreatic cancer cells. PANC-1 and BxPC-3 cells were pretreated with either 200 nM or 500 nM rapamycin for 24 h, and then treated with 1 µM GA for another 24 h. Cell viability was detected by the MTT assay. Data are presented as mean ± SD (n = 3); ** indicates P < 0.01, *** indicates P < 0.001. [file 12935_2018_705_MOESM2_ESM.jpg]
